# Supplementary material for: Preoperative Inflammatory Markers and the Risk of Postoperative Delirium in Patients Undergoing Lumbar Spinal Fusion Surgery
Source: J Clin Med. 2022 Jul 14;11(14):4085. doi: 10.3390/jcm11144085 (PMC9324332; doi:10.3390/jcm11144085)
Supplement: Supplementary file 1 [file jcm-11-04085-s001.zip › jcm-1811184-supplementary.pdf]

Table S1. Predetermined word list describing delirium.

Actually investigated words	Translation in English
delirium	delirium
confusion	confusion
psychosis	psychosis
violent	violent
vigilant	vigilant
hyper alert	hyper alert
attention	attention
irritable	irritable
attentional deficit	attentional deficit
fluctuation	fluctuation
섬망	delirium
헛소리	gibberish, delirious talking
헛것	visual hallucination
지른다	yell
환시	visual hallucination
환청	auditory hallucination
환각	hallucination
망상	delusion
의식 변화	altered mentality
의식 혼탁	clouding of consciousness
집중력	concentration
외부 자극	external stimuli
외부 반응	response to a stimulus
반응성	response
이상행동	abnormal behavior
과다행동	hyperactive
행동변화	behavioral changes
공격	aggression
난폭	violence
충동적	impulsive
돌발행동	unexpected behavior
집중력	concentration
주의산만	distraction
횡설수설	gibberish
안절부절	restlessness
불안	restlessness
엉뚱	absurd
비논리적	irrational
상관없는	unconnected
각성	alertness
무기력	lethargy
인지능력	cognitive function

초조	restlessness
흥분	agitation
혼미	confusion
혼돈	confusion
혼란	confusion
지남력	orientation
불면	insomnia
요실금	urinary incontinence
졸림	drowsy
배회	wandering
혼잣말	self-talking
욕설	swear
고함	yell

Table S2. Drugs of Medication Factors

Pharmacologic category	Drug
CCBs	Amlodipine, Barnidipine, Benidipine, Cilnidipine, Diltiazem, Efonidipine, Felodipine, Lacidipine, Lercanidipine Manidipine, Nicardipine, Nifedipine, Verapamil
Diuretics	Acetazolamide, Amiloride, Furosemide, hydrochlorothiazide, D-mannitol, Indapamide, Isosorbide, Spironolactone, Torasemide
Beta blockers	Arotinolol, Atenolol, Bisoprolol, Carvedilol, Labetalol, Metoprolol, Nebivolol, Propranolol
ACE inhibitors	Captopril, Cilazapril, Enalapril, Fosinopril Perindopril, Ramipril
ARBs	Candesartan, Eprosartan, Fimasartan Irbesartan, Losartan, Olmesartan, Telmisartan, Valsartan
Other antihypertensives	Amlodipine/Losartan, Ambrisentan, Bisoprolo/Hydrochlorothiazide, Bosentan. Candesartan/ Hydrochlorothiazide, Eprosartan/Hydrochlorothiazide, Felodipin/Ramipril, Fimasartan/Hydrochlorothiazide, Irbesartan/Hydrochlorothiazide, Lercanidipine/Valsartan, Losartan /Hydrochlorothiazide, Amlodipine/Olmesartan/Hydrochlorothiazide, Amlodipine/Olmesartan, Olmesartan/Hydrochlorothiazide, Sildenafil, Amlodipine/Telmisartan, Telmisartan/ Hydrochlorothiazide, Amlodipine/Valsartan, Valsartan/ Hydrochlorothiazide
Miscellaneous CV drugs	Amlodipine/Atorvastatin, Irbesartan/Atorvastatin, Ulinastatin, Ivabradine, Olmesartan/Rosuvastatin, Omega-3 acid ethyl esters, Telmisartan/Rosuvastatin, Trimetazidine, Pitavastatin/Valsartan
Antidepressants	Amitriptyline, Amoxapine, Doxepin, Imipramine, nortriptyline, Tianeptine, Fluvoxamine, Paroxetine, Escitalopram, Sertraline, Vortioxetine, Bupropion, Mirtazapine, Trazodone, Duloxetine, Desvenlafaxine,

	Milnacipran, Venlafaxine
Hypnotics & sedatives	Alprazolam, Buspirone, Chlordiazepoxide, Chlordiazepoxide/Clidinium, Clobazam Chloral, Diazepam, Etizolam, Flurazepam, Dexmedetomidine, Lorazepam, Midazolam, Phenobarbital, Melatonin, Tandospirone, Triazolam, Zolpidem
Antipsychotics	Amisulpride, Aripiprazole, Blonanserin, Clozapine, Chlorpromazine, Haloperidol, Paliperidone, Risperidone, Olanzapine, Paliperidone, Quetiapine, Sulpiride, Ziprasidone
CNS stimulants	Atomoxetine, Clonidine, Modafinil, Methylphenidate
Antimanic agents	Lithium
Opioids	Buprenorphine, Fentanyl, Morphine, Oxycodone, Pethidine Sufentanil, Hydromorphone, Oxycodone,
Other analgesics (no NSAIDs)	Acetaminophen, Capsaicin, Ethanol, Nefopam, Propacetamol, Tramadol
NSAIDs	Aceclofenac, Celecoxib, Dexibuprofen, Diclofenac, Etoricoxib, Ibuprofen, Imidazole salicylate, Indomethacin, Ketoprofen, Ketorolac, Loxoprofen, Meloxicam, Nabumetone, Naproxen, Pelubiprofen, Polmacoxib, Zaltoprofen
Skeletal muscle relaxants	Afloqualone, Baclofen, Cyclobenzaprine, Dantrolene, Eperisone, Clostridium botulinum A toxin, Cisatracurium, Gallamine, Rocuronium, Suxamethonium, Vecuronium, Orphenadrine, Chlorphenesin, Thiocolchicoside, Aescin, Tizanidine
Corticosteroids	Budesonide, Deflazacort, Dexamethasone, Fludrocortisone, Hydrocortisone, Methylprednisolone, Triamcinolon, Prednisolone
Antiplatelet agents	Aspirin, Cilostazol, Cilostazol/Ginkgo Biloba Leaf Extract, Clopidogrel, Clopidogrel/Aspirin, Abciximab, Prasugrel, Sulodexide, Ticlopidine, Ticagrelor, Triflusal
Anticoagulants	Human Antithrombin Ⅲ, Enoxaparin, Gabexate, Heparin, Tirofiban, Warfarin, Tinzaparin, Apixaban, Dabigatran, Edoxaban, Argatroban, Rivaroxaban
Antihyperlipidemic agents	Atorvastatin/Ezetimibe, Atorvastatin, Cholestyramine, Ezetimibe, Fenofibric acid, Fluvastatin, Gemfibrozil, Lovastatin, Pitavastatin, Pravastatin/Fenofibrate, Rosuvastatin, Rosuvastatin/Ezetimibe, Simvastatin/ Ezetimibe, Simvastatin/Fenofibrate, Simvastatin
Antihistamines/antiallergics	Azelastine, Levocetirizine, Chlorpheniramin, Dimenhydrinate, Desloratadine, Ebastine, Pseudoephedrine, Emedastine, Epinastine, Fexofenadine, γ-Linolenic acid, Hydroxyzine, Chlorpheniramine, Human IgG/Histamine, Ketotifen, Purified House Dust Mite allergen extract, Loratadine, Mequitazine, Olopatadine, Allergen extracts, Tranilast
H2 receptor antagonist	Cimetidine, Famotidine, Lafutidine, Nizatidine, Ranitidine

CCBs, Calcium channel blockers; ACE inhibitors: Angiotensin Converting Enzyme Inhibitors; ARBs, Angiotension II Receptor Blockers; CV, cardiovascular; NSAIDs, non-steroidal anti-inflammatory drugs.

Table S3. Definition of each variable

Variables	Definition
**Preoperative variables**	
Age	Age at surgery
Obesity (BMI > 29.9)	Obesity was defined as a body mass index greater than 29.9.
ASA physical status > 2	Preoperative scores for American Society of Anesthesiology physical status classification system greater than 2
Emergency surgery	Surgery performed as an emergency
HTN	Patients with hypertension as a preoperative comorbidity<br>Patient group classified by hypertension diagnostic code or recorded on admission notes
DM	Patients with DM as a preoperative comorbidity<br>Patient group classified by DM diagnostic code or recorded on admission notes
Heart disease	Patients with heart disease as a preoperative comorbidity<br>Patient group classified by heart disease diagnostic code or recorded on admission notes
Stroke	Patients with stroke as a preoperative comorbidity<br>Patient group classified by stroke diagnostic code or recorded on admission notes
Cancer	Patients with cancer as a preoperative comorbidity<br>Patient group classified by cancer diagnostic code or recorded on admission notes
Dyslipidemia	Patients with dyslipidemia as a preoperative comorbidity<br>Patient group classified by dyslipidemia diagnostic code or recorded on admission notes
Parkinson's disease	Patients with Parkinson's disease as a preoperative comorbidity<br>Patient group classified by Parkinson's disease diagnostic code or recorded on admission notes
Dementia	Patients with dementia as a preoperative comorbidity<br>Patient group classified by dementia diagnostic code or recorded on admission notes
Depression	Patients with depression as a preoperative comorbidity<br>Patient group classified by depression diagnostic code or recorded on admission notes
Kidney disease	Patients with kidney disease as a preoperative comorbidity<br>Patient group classified by kidney disease diagnostic code or recorded on admission notes
Liver disease	Patients with liver disease as a preoperative comorbidity<br>Patient group classified by liver disease diagnostic code or recorded on admission notes
Insomnia	Patients with insomnia as a preoperative comorbidity<br>Patient group classified by insomnia diagnostic code or recorded on admission notes
Sleep disorder	Patients with sleep disorder as a preoperative comorbidity<br>Patient group classified by sleep disorder diagnostic code or recorded on admission notes
Alcohol	Patients with a history of drinking alcohol more than once per week at the time of preoperative assessment
Smoking	Current smoker at the time of preoperative assessment
**Intra and postoperative variables**	
Postop. ICU care	Patients receiving intensive care in the ICU after surgery
Patient-controlled analgesia	Patients using a patient-controlled pain therapy device after surgery
Operation time	Total time of the surgery

Surgical range	The number of vertebrae involved in the lumbar spinal fusion surgery
Fluid balance	Fluid volume input minus output during surgery
